# Reproducibility of coronary vessel wall imaging techniques

**DOI:** 10.1186/1532-429X-13-S1-O11

**Published:** 2011-02-02

**Authors:** Andrew D Scott, Jennifer Keegan, David N Firmin

**Affiliations:** 1 Imperial College London, London, UK; 2 The Royal Brompton & Harefield NHS Foundation Trust, London, UK

## Objective

To evaluate the reproducibility of three coronary artery wall imaging techniques.

## Background

Coronary wall thickness measurements must be highly reproducible to be useful in longitudinal studies. While 2D turbo-spin-echo (TSE) and spiral techniques are commonly used, 3D techniques reduce partial-volume effects and allow greater vessel coverage. Recently, 3D coronary wall imaging was demonstrated with 100% respiratory efficiency (RE) using beat-to-beat respiratory motion correction (B2B-RMC)[1] which uses motion information determined from low resolution 3D images of the fat around the vessel as a surrogate for vessel motion. Here we assess the reproducibility of this technique together with that of navigator-gated 2D TSE and spiral acquisitions.

## Methods

Cross-sectional right coronary artery wall images were obtained in 10 healthy subjects on a Siemens 1.5T Avanto scanner using dark-blood prepared B2B-RMC 3D spiral imaging, navigator-gated 2D TSE imaging and navigator-gated 2D spiral imaging. Acquisition order was randomized and subjects were imaged on two separate occasions to assess inter-study reproducibility using the intra-class correlation-coefficient (ICC) and Bland Altman analysis. All acquisitions used 0.7x0.7mm in-plane resolution. B2B-RMC 3D spiral acquisitions acquired 8x3.0mm slices (16x1.5mm reconstructed) and 2D techniques acquired 1x6mm slice. Navigator-gated techniques used a 5mm window while B2B-RMC excluded data acquired at only very extreme respiratory positions, retrospectively correcting the rest. Durations, assuming 100% RE, were 600 cardiac cycles (CC) for B2B-RMC, 75CC for 2D spiral and 202-576CC for 2D TSE acquisitions depending on echo-train length and phase oversampling.

A single slice was selected from each 3D acquisition for comparison with the 2D acquisitions. For all acquisitions, average wall thickness was obtained from circles drawn around the outer and inner edges of the vessel wall. The intra- and inter-observer reproducibility of this measurement technique was analysed in 20 images.

## Results

**Figure 1:**
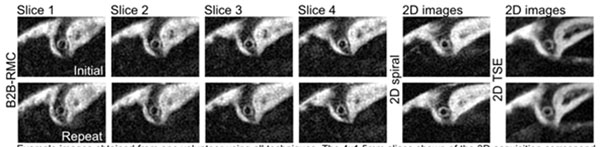
Example images obtained from one volunteer using all techniques. The4x1.5mm slices shown of the 3D acquisition correspond to the single 6mm slices of the 2D acquisitions. There is a high degree of visual similarity between the initial (upper) and repeat images (below) acquired 29 days apart. Respiratory efficiency in the initial studies was 100% B2B-RMC, 40% 2D spiral and 38* TSE. In the repeat studies respiratory efficiency was 100% B2B-RMC, 55% 2D spiral and 40% 2D TSE.

Example images from one subject are shown in figure 1. 92% of acquisitions were successful. RE, wall thickness and acquisition durations are presented in Table 1. B2B-RMC RE was less variable and significantly higher than navigator gating (99.6±1.2%vs.39.0±7.5%,p<0.0001) and there was no significant difference in vessel wall thickness between techniques(p=ns).

**Table 1:** Results of the comparison between the three coronary vessel wall imaging techniques.

Method	3D spiral B2B-RMC	2D spiral navigator	2D TSE navigator
Respiratory efficiency (%)	99.5±1.6	39.0±9.4	39.3±4.6
Total duration (RR-intervals)	603±10	209±59	479±74
Acquisition duration/reconstructed slice (RR-intervals)	37.7±0.6	209±59	479±74
Wall thickness (mm)	1.10±0.14	1.14±0.15	1.21±0.17
N	9	10	8

Reproducibility data are presented in Table 2. The B2B-RMC technique was most reproducible (ICC 0.84, mean difference 0.01±0.10mm). Mean intra-/inter-observer wall thickness difference was 0.04±0.09mm/0.05±0.08mm.

**Table 2:** Reproducibility of the three coronary vessel wall imaging techniques.

Method	3D spiral B2B-RMC	2D spiral navigator	2D TSE navigator
Mean wall thickness difference (initial-repeat acquisition) (mm)	0.01±0.10	-0.01±0.14	0.06±0.14
Intra-class correlation	0.86	0.70	0.72
N	9	10	6

## Conclusions

ICCs were good for the 2D techniques and excellent for the 3D technique. The high RE of B2B-RMC enables reproducible 3D coronary wall assessment within a reasonable duration which will permit improved assessment of atherosclerotic disease.
